# Hepatic Tumor Rupture in Other Iatrogenic Immunodeficiency-Associated Lymphoproliferative Disorders of the B-cell Type in a Patient With Chronic Rheumatoid Arthritis

**DOI:** 10.7759/cureus.56615

**Published:** 2024-03-21

**Authors:** Kazuto Togitani, Yoshiki Uemura, Hiroshi Sakaeda

**Affiliations:** 1 Department of Hematology, Chikamori Hospital, Kochi, JPN; 2 Department of Gastroenterology, Chikamori Hospital, Kochi, JPN

**Keywords:** r-chop chemotherapy, diffuse large b-cell hepatic lymphoma, transcatheter arterial embolization, iatrogenic immunodeficiency-associated lymphoproliferative disorders, hepatic malignant lymphoma

## Abstract

A 75-year-old woman on tumor necrosis factor inhibitors for rheumatoid arthritis presented with hematemesis and a gastric biopsy revealed diffuse large B-cell lymphoma with possible bulky left liver tumor involvement. On the second day of treatment with rituximab, cyclophosphamide, doxorubicin, vincristine, and prednisone, the patient experienced abdominal pain followed by shock vitals. A contrast-enhanced computed tomography scan revealed a ruptured liver. Transcatheter arterial embolization (TAE) was performed to stop the bleeding. This is the first case of hepatic tumor rupture secondary to an iatrogenic immunodeficiency-associated lymphoproliferative disorder of the B-cell type that was successfully treated with TAE to achieve hemostasis.

## Introduction

Diffuse large B-cell lymphoma (DLBCL) is the most common form of aggressive non-Hodgkin lymphoma (NHL), accounting for 30% of cases [1]. In up to 40% of cases, the disease arises in extranodal tissues [2]. The most common site of primary extranodal disease is the stomach/gastrointestinal tract [2]. Primary hepatic lymphoma is rare (0.016% of all NHLs), but secondary involvement in the liver is commonly observed in about 20% of cases [3].

Although rupture of hepatic tumor is a common event in hepatocellular carcinoma (HCC), with the incidence reported to be approximately 10% in Japan [4], hepatic lymphoma leading to rupture is an extremely rare complication that has been described in only three cases thus far [5-7].

Other iatrogenic immunodeficiency-associated lymphoproliferative disorders (OII-LPDs) are lymphoid proliferations or lymphomas that arise in patients treated with immunosuppressive drugs for autoimmune disease [8]. Almost half of OII-LPD patients have extranodal disease, in which the liver has been reported to be the most frequently affected location [9]. Recently, a case of primary hepatic OII-LPD after methotrexate (MTX) administration was reported with a large liver tumor showing rapid growth, but the patient was doing well on chemotherapy [10].

Here, we report the first case of OII-LPD of the B-cell type that presented with a rupture of the hepatic tumor which was then successfully treated with transcatheter arterial embolization (TAE), to obtain hemostasis, and systemic chemotherapy. In addition, the clinical characteristics of cases of hepatic lymphoma with tumor rupture in the literature are discussed.

## Case presentation

**Figure 1:**
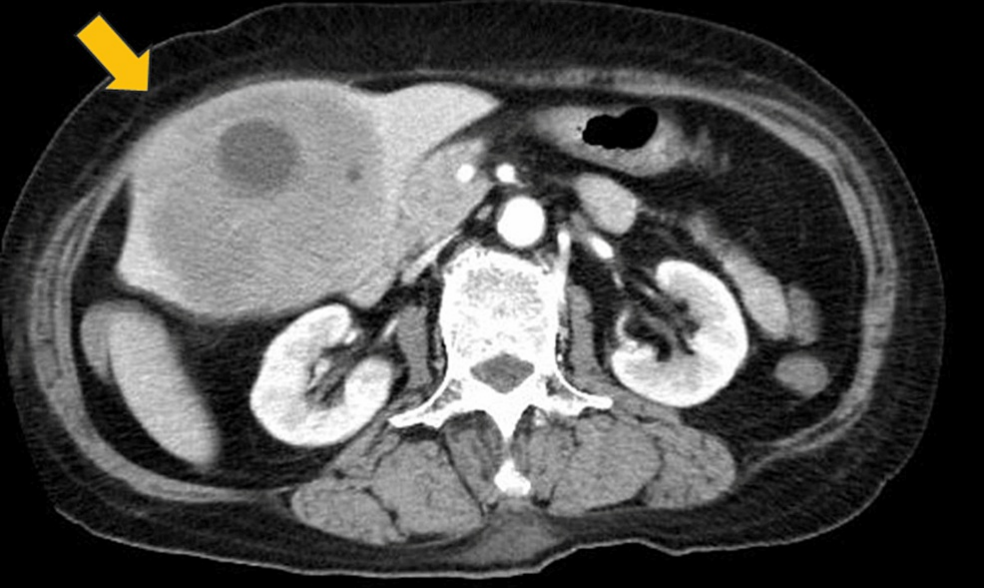
Contrast-enhanced computed tomography scan showed a huge hypoenhancing tumor in the left liver with a bulging, exophytic contour (arrow).

A 75-year-old woman was diagnosed with rheumatoid arthritis five years prior and had been treated with MTX for two years. Due to poor response starting from just less than three years previously, MTX was stopped and she was started on iguratimod and tumor necrosis factor α inhibitors (TNFis). Initially, abatacept was used, but was discontinued due to skin rash, and golimumab was subsequently prescribed. Three months previously, she had visited her local otolaryngologist for nasal obstruction and was scheduled for surgery for sinusitis. Three weeks earlier, the patient was brought to the emergency room of another hospital due to hematemesis. Urgent endoscopy revealed a bleeding gastric ulcer, and clipping was performed to achieve hemostasis. A whole-body computed tomography (CT) scan showed a tumor in the paranasal cavity and a bulky tumor in the left liver (111 x 77 mm) (Figure 1).

Hematoxylin and eosin staining of an obtained gastric biopsy specimen showed that the gastric submucosa had been replaced by diffuse lymphoid cells. An immunohistochemical analysis of abnormal lymphoid cells revealed positivity for CD20, CD79a, MIB-1 (70%), B-cell lymphoma (BCL)2, BCL6, and MUM1 and negativity for CD3, CD10, and Epstein-Barr encoding region in situ hybridization. Thus, the histological findings showed a non-germinal, center-type DLBCL pattern. Based on the patient’s history of receiving MTX and TNFi, she was diagnosed with OII-LPD of the B-cell type. Despite the withdrawal of golimumab, the liver mass was growing so rapidly that the patient was referred to our hospital. Vital signs at the time of transfer were a blood pressure of 116/58 mmHg, pulse rate of 107 beats/minute, and temperature of 37.5°C. Laboratory data showed increased neutrophils, mild anemia, and thrombocytopenia (Table 1), biochemical tests showed elevated C-reactive protein and biliary obstructive liver damage (Table 2), and serological tests showed elevated soluble interleukin-2 receptor, carbohydrate antigen 19-9 and protein induced by vitamin K absence II; however, alfa-fetoprotein and carcinoembryonic antigen were normal, hepatitis B and C were negative (Table 3), and there was no history of cirrhosis. Hence, HCC was considered negative.

**Table 1:** Patient’s blood analysis on admission. WBC = white blood cell; RBC = red blood cell; MCV = mean corpuscular volume; PT = prothrombin time; INR = international normalized ratio; APTT = activated partial thromboplastin time; FDP = fibrin degradation product

	Patient’s result	Normal range
WBC	16,400	3,300–8,600/µL
Band	0%	3–6%
Segment	81%	45–50%
Lymphocyte	5%	25–45%
Monocyte	14%	4–7%
Eosinophil	0%	1–4%
Basophil	0%	0–1%
RBC	335	386–492 × 10^4^/µL
Hemoglobin	9.1	11.6–14.8 g/dL
Hematocrit	28.3	35.1–44.4%
MCV	84.5	83.6–98.2 fL
Platelet count	12.8	15.8-34.8 × 10^4^/µL
PT	14.1	10–13 seconds
PT-INR	1.23	1
APTT	42.8	20–40 seconds
Fibrinogen	428.9	180–350 mg/dL
FDP	7.3	<5.0 µg/mL
D-dimer	2	<1.0 µg/mL

**Table 2:** Patient’s biochemical analysis on admission. TP = total protein; Alb = albumin; AST = aspartate transaminase; ALT = alanine transaminase; LDH = lactate dehydrogenase; ALP = alkaline phosphatase; γGTP = gamma-glutamyl transferase; CHE = cholinesterase; T-Bil = total bilirubin; D-Bil = direct bilirubin; Amy-S = serum amylase; Crn = serum creatinine; BUN = blood urea nitrogen; UA = uric acid; Na = serum sodium; K = serum potassium; Cl = serum chloride; Ca = serum calcium; T-Cho = total cholesterol; CRP = C-reactive protein; Glu = blood glucose; HbA1c = hemoglobin A1c

	Patient’s result	Normal range
TP	4.6	6.6–8.1 g/dL
Alb	2.1	4.1–5.1 g/dL
AST	90	13–30 U/L
ALT	56	7–23 U/L
LDH	565	124–222 U/L
ALP	645	38–113 U/L
γGTP	367	9–32 U/L
CHE	114	201–421 U/L
T-Bil	2	0.4–1.5 mg/dL
D-Bil	1.4	0–0.3 mg/dL
Amy-S	31	44–132 U/L
Crn	0.72	0.46–0.79 mg/dL
BUN	12.3	8–20 mg/dL
UA	5.9	2.6–5.5 mg/dL
Na	131	138–145 mEq/L
K	3.9	3.6–4.8 mEq/L
Cl	98	101–108 mEq/L
Ca	7.2	8.8–10.1 mEq/L
T-Cho	104	142–248 mg/dL
CRP	19.37	0–0.14 mEq/L
Glu	142	73–109 mEq/L
HbA1c	4.4	4.6–6.2%

**Table 3:** Patient’s serological analysis on admission. AFP = alpha-fetoprotein test; CEA = carcinoembryonic antigen; CA 19-9 = carbohydrate antigen 19-9; PIVKA-II = protein induced by vitamin K absence-II; sIL2-R = soluble IL-2 receptor α; HBsAg = hepatitis B surface antigen; HBs Ab = hepatitis B surface antibody; HBc Ab = hepatitis B core antibody; HCV Ab = hepatitis C virus antibody

	Patient’s result	Normal range
AFP	3.2	<10.0 ng/mL
CEA	0.9	<5.0 ng/mL
CA 19-9	594.4	<37.0 U/mL
PIVKA-II	163	<40 mAU/mL
sIL2-R	12,200	157–474 U/mL
Ferritin	7,822.2	3.6–114 ng/mL
HBsAg	(-)	(-)
HBs Ab	(-)	(-)
HBc Ab	(-)	(-)
HCV Ab	(-)	(-)

**Figure 2:**
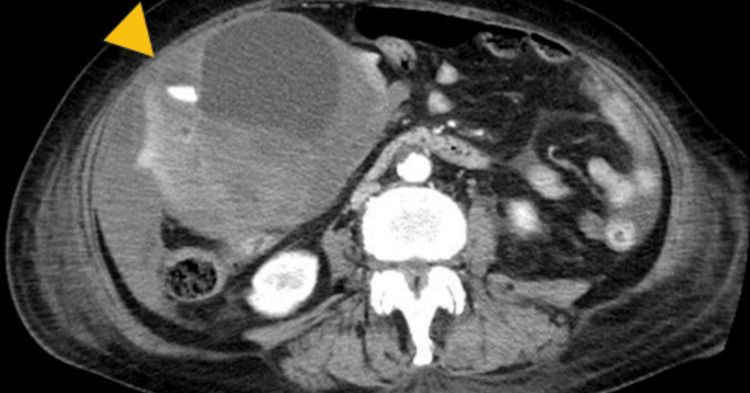
Contrast-enhanced computed tomography scan showing intratumoral extravasation (arrowhead) during the arterial phase and hemoperitoneum due to rupture of the tumor.

From these findings, it was thought that the patient was experiencing an exacerbation of hepatic lymphoma with gastric, paranasal, and para-aortic lymph nodal involvement. Prognostic factors indicated high risk according to the International Prognostic Index (IPI). There was no time window to perform a liver tumor biopsy. Hence, chemotherapy was administered first. At midnight on the day after initiation of rituximab, cyclophosphamide, doxorubicin, vincristine, and prednisone (R-CHOP) treatment, the patient complained about abdominal pain and appeared pale. Incontinence and hypotension (blood pressure 80/33) were also noted and laboratory data showed a decrease in hemoglobin (Hb) concentration (Hb: 6.9 g/dL). Repeated CT showed rupture of the liver tumor with intratumoral hemorrhage and hemoperitoneum (Figure 2).

**Figure 3:**
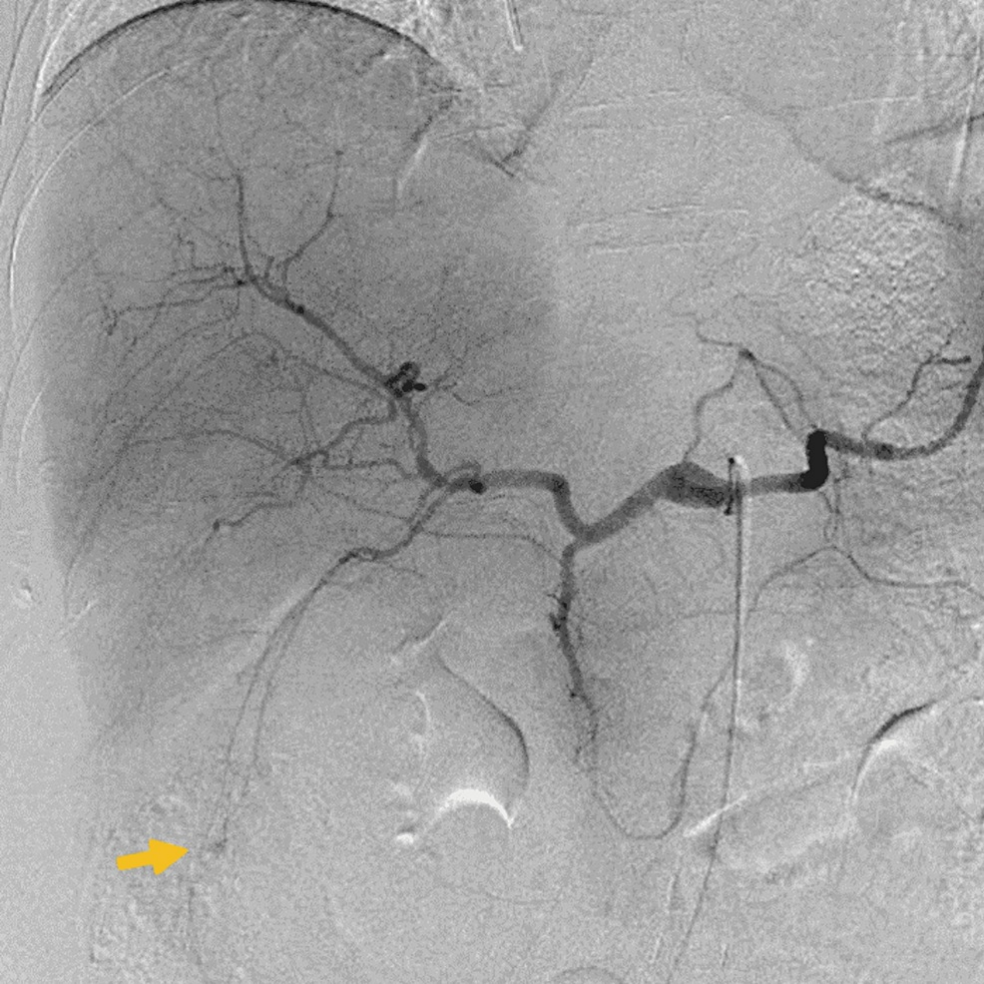
Selective hepatic arteriogram from the left lateral artery (A3) showing extravasation of the contrast medium (arrow).

Selective hepatic arteriogram from the left lateral artery (A3) showed extravasation of contrast medium (Figure 3).

TAE from A3 was performed and led to successful hemostasis. The progression of anemia halted, and over the next three weeks, the right hypochondoralgia and nasal obstruction gradually improved. The patient received eight cycles of R-CHOP chemotherapy. Concurrently, we also prescribed prophylactic intrathecal methotrexate, cytarabine, and dexamethasone for eight doses in accordance with the patient’s high-risk status according to the central nervous system (CNS)-IPI. Subsequently, the patient achieved complete remission which has continued for 11 months.

## Discussion

Rupture of hepatic tumors is a common event in HCC, with the incidence reported to be approximately 10% in Japan [7]. In contrast, hepatic rupture in malignant lymphoma is extremely rare. There have been only four cases reported, including the present case (Table 4).

**Table 4:** Summary of hepatic tumor rupture cases in malignant lymphoma. DLBCL = diffuse large B-cell lymphoma; non-GCB = non-germinal center B-cell; Cx = chemotherapy; CPT-11 = irinotecan; R-CVP = rituximab, cyclophosphamide, vincristine, and prednisolone; IT = intrathecal chemotherapy; CECT = contrast-enhanced computed tomography; Tx = therapy; TAE = transcatheter arterial embolization; CR = complete remission; CNS = central nervous system

Case number	Age/Sex	Histology	Location	Size (maximum)	Prior Cx	Day of Cx	Clinical presentation	Diagnosis	Tx of Hematostasis/Outcome	Outcome/Follow-up	Reference
1	60/M	DLBCL	S8	8 cm	CPT-11	19	Shock, abdominal pain	Autopsy	Conservative /Failure	Death from bleeding/<1 month	Tsutani et al., 1999 [5]
2	74/M	DLBCL	S6	9.4 cm	none	-42	Right hypochondrial pain	CECT	TAE/Success	CR/30 months	Oshima et al., 2018 [6]
3	51/F	DLBCL, non-GCB type	S6	13 cm	R-CVP	6	Tachycardia, anemia	CECT	Conservative/Success	Death from CNS relapse/18 months	Segawa et al., 2022 [7]
4	75/F	DLBCL, non-GCB type	S3	11.1 cm	R-CHOP	2	Abdominal pain, shock	CECT	TAE/Success	CR/15 months	Present case

Among these, two cases originated from segment S6 and one (our case) from segment S3, suggesting that, as with HCC, tumors originating from certain smaller liver segments, such as the caudate lobe (segment S1), the left lateral segments (segments S2 and S3), and the right posterior-inferior segment (segment S6), may be more prone to rupture (the small room hypothesis noted by Sahu et al. [4]). Moreover, rapid and exophytic growth of the tumor in the present case (Figure 1) and Case 1 in Table 4 (which showed exophytic and sub-capsular growth) could lead to increased intratumoral pressure and consequent rupture. In the remaining three cases except for Case 2, liver tumor rupture occurred 2-19 days after chemotherapy, suggesting the influence of chemotherapy even in hepatic lymphoma as in transarterial chemoembolization for HCC [11,12].

The present patient had discontinued MTX for 2.5 years before the onset of lymphoma and was receiving TNFi instead. Although previous studies have shown inconsistent results regarding the association between the use of TNFi and the development of hematologic malignancies in patients with autoimmune diseases, recent studies have demonstrated an increased risk of lymphoma over the 1.5-2.5 years for which TNFis were prescribed [13,14]. Therefore, a correlation between TNFi and lymphoma development in this patient cannot be excluded.

Among hepatic rupture cases in malignant lymphoma (Table 4), Case 3 eventually developed CNS recurrence and had an unfortunate course. Referring to a case report and the present’s patient high-risk status on the CNS-IPI [15], she was treated with intrathecal chemotherapy as a CNS prophylactic measure and had no CNS recurrence at 11 months post-treatment.

## Conclusions

Tumor rupture in hepatic lymphoma is a rather rare event compared to the same occurrence in HCC but should be noted in cases of large masses arising in small liver segments showing an exophytic growth pattern, especially in the first few days after initiation of chemotherapy. In rheumatoid arthritis patients receiving TNFi for a couple of years, the development of lymphoma should be noted in the presence of liver function abnormalities and liver masses.
